# Effects of Light Pedaling Added to Contrast Water Immersion for Recovery after Exhaustive Exercise

**DOI:** 10.3390/ijerph182413068

**Published:** 2021-12-11

**Authors:** Gaelle Deley, Carole Cometti, Christos Paizis, Nicolas Babault

**Affiliations:** 1 INSERM Unity 1093-Cognition Action Plasticité Sensorimotrice, Université Bourgogne Franche-Comté, Faculté des Sciences du Sport, F-21000 Dijon, France; carole.cometti@u-bourgogne.fr (C.C.); christos.paizis@u-bourgogne.fr (C.P.); nicolas.babault@u-bourgogne.fr (N.B.); 2 Centre d’Expertise de la Performance, Université de Bourgogne Franche-Comté, Faculté des Sciences du Sport, F-21000 Dijon, France

**Keywords:** blood lactate concentration, perceived exertion, performance, contrast water immersion, combined recovery, vertical jump

## Abstract

For years, athletes and coaches have been looking for new strategies to optimize post-exercise recovery; it has recently been suggested that combining several methods might be a great option. This study therefore aimed to investigate the efficacy of contrast water therapy (CWT) used alone or associated with pedaling to recover from exhaustive exercise. After high-intensity intermittent exercise, 33 participants underwent 30 min of either (i) passive rest (PASSIVE), (ii) CWT with pedaling while in water (COMB) or (iii) classic CWT (CWT). Blood lactate concentration, countermovement jump height and perceived exhaustion were recorded before exercise, immediately after, after recovery interventions and after an additional 30 min of passive rest. Blood lactate concentration returned to initial values after 30 min of COMB (5.9 mmol/L), whereas in the other conditions even 60 min was not enough (10.2 and 9.6 mmol/L for PASSIVE and CWT, respectively, *p* < 0.05). Jump height was close to initial values after 30 min of CWT (37.3 cm), whereas values were still depressed after 60 min in the PASSIVE (36.0 cm) and COMB (35.7 cm) conditions (*p* < 0.05). Perceived exertion was still high for all conditions after 60 min. The present results are in favor of the utilization of CWT after exhaustive exercise, but the modality has to be chosen depending on what comes next (subsequent exercise scheduled in the following hours or further away).

## 1. Introduction

A common issue confronting high-level athletes is the great amount of exercise (training, competition) they must undertake and the limited time available for recovery [1]. This may lead to fatigue accumulation as the season progresses and cause symptoms of over-reaching, such as exhaustion, resulting in performance decreases [1]. For these reasons, finding strategies to optimize recovery and accelerate performance restoration is a big challenge to which various interventions have been proposed, with mitigated results [2,3,4,5]. These modalities include active recovery, compression garments, electrical stimulation, stretching, sauna, massage, water immersions, etc., and their use often depends on athletes’ habits and levels [6,7]. In particular, water immersion is frequently used in team sports [6].

Among water immersion strategies, we can distinguish thermoneutral water immersion, cold water immersion, hot water immersion and contrast water therapy (CWT, alternating immersions in cold and hot water). Cold water immersion and CWT have been more extensively studied in the literature than the other two, but to date there is no consensus regarding their effectiveness. While cold water immersion is supposed to have beneficial effects due to temperature-induced vasoconstriction, CWT is thought to accelerate recovery thanks to “vaso-pumping” [8]. More specifically, the alternation of vasodilatation and vasoconstriction of the blood vessels due to temperature changes [9] might create fluctuations in blood flow, therefore enhancing the removal of metabolic by-products and consequently speeding-up recovery [10,11,12]. However, despite being widely used by athletes and coaches [6], scientific evidence regarding this method is still missing: although most studies revealed positive effects of CWT on perceptual recovery [13,14], its effects on performance are less clear [13,15]. For Wilcock et al. [8], this absence of clear beneficial effects might be explained by the frequency of the vaso-pumping induced by CWT (0.03–0.008 Hz), which appears to be too low to have significant effects on blood flow. Conversely, with muscular pumping occurring at a rate around 2 Hz, active recovery has often been demonstrated to be more effective for recovery than passive modalities [16]. Indeed, low-intensity repetitive contraction–relaxation (such as light pedaling) may have beneficial effects for recovery thanks to increased muscle blood flow [17]. Therefore, adding light pedaling during CWT might be of great interest to maximize recovery, particularly after exercise that induces great increases in metabolic by-products [17].

As suggested by Barnett [18], recovery should not be confined to a single method; a great option might be to combine recovery strategies. Recently, Crowther et al. [13] evaluated the effects of several recovery strategies, one of which was combined cold water immersion and active recovery. However, to our knowledge, no study has investigated the effects of combining CWT and active recovery. Therefore, the main aim of the present study was to compare the effects of a recovery period in passive conditions (PASSIVE), using contrast water immersion alone (CWT) or combining CWT with light pedaling (COMB). Comparisons were made on blood lactate concentration, countermovement jump performance (CMJ) and the rate of perceived exhaustion (RPE). We hypothesized that adding light pedaling, known to favor blood flow [16], during water immersion would maximize the effects of recovery after exhaustive intermittent exercise known to increase blood lactate concentration [19]. 

## 2. Materials and Methods

Thirty-three well-trained male team-sport players (age 21.4 ± 1.1 years, height 176.0 ± 9.0 cm, body mass 68.9 ± 13.9 kg and 6.4 ± 2.6 h of physical activity per week) agreed to take part in the study. The inclusion/exclusion criteria were: healthy; male between 18 and 30 years old; practicing team sport at least 4 h/week; presenting normal hip, knee and ankle joint range of motion; not taking pain killers, tranquilizers or antidepressants; not presenting skeletal muscle dysfunction in the lower extremities; and not presenting any cardiovascular or peripheral vascular disorders, chronic diseases or neurological or muscular dysfunctions. Participants were all instructed to refrain from training 48 h before testing. 

### 2.1. Experimental Design

Prior to the experiment, participants were randomly allocated to (i) passive rest (PASSIVE, n = 12), (ii) contrast water therapy with pedaling while in water (COMB, n = 12) or (iii) classic contrast water therapy (CWT, n = 9) recovery conditions (each subject only performed one condition). 

**Figure 1:**
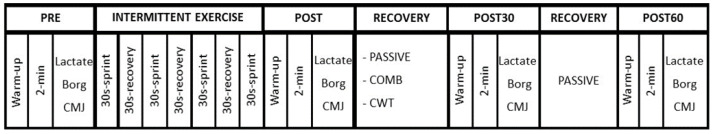
Schematic view of a testing session. CMJ: Counter Movement Jump, PASSIVE: subjects remained passively seated, COMB: light pedalling in both hot and cold water and CWT: subjects remained passively seated in hot and cold water alternatively. PRE: tests performed before the exhaustive exercise, POST: tests performed immediately after the exhaustive exercise, POST30: performed 30 min after the exhaustive exercise, POST60: performed 60 min after the exhaustive exercise.

An overview of the experimental protocol is presented in Figure 1. Briefly, all participants completed a standardized 10-min warm-up followed by 2 min of passive recovery [20]. Afterwards, blood lactate concentration, perceived exertion (RPE) and vertical jump performance were measured; exhaustive intermittent exercise was then performed. This fatiguing exercise was immediately followed by the same measurements (POST). After this, participants had a 60 min recovery period, with tests performed after 30 min (POST30) and at the end of the 60 min (POST60).

### 2.2. Exhaustive Intermittent Exercise

The exercise consisted of four 30-s sprints separated by 30 s of passive rest, performed on a mechanically braked cycle ergometer (CycleOps Power, 300 Pro, Saris Cycling Group, Madison WI, USA) with a preset load of 0.075 × body mass (Kg) [20]. All participants received verbal encouragement throughout the 30-s pedaling periods. This protocol was chosen to invoke metabolic and local muscular fatigue [19,21,22].

This exercise was preceded by a standardized warm-up consisting of three 30-s intervals of pedaling against an increasing resistance of 25, 50 and 75% of the test load alternated with 30-s intervals of active rest (pedaling against no resistance at 80 rpm). Prior research has indicated that this form of pre-test loading elicits optimal power production during supramaximal exercises [20]. The bicycle’s saddle height was adjusted individually to allow appropriate knee extension with ankles flexed at 90°.

### 2.3. Measurements

Maximal, mean and minimal power values were measured during the first and fourth periods of exhaustive intermittent exercise in order to quantify fatigue.

***PRE and POST exhaustive exercise:*** As mentioned above, measurements performed before (PRE) and immediately after (POST) the exhaustive intermittent exercise included (i) fingertip blood draws to measure blood lactate concentration response to exercise, (ii) a 10-point Borg scale to quantify the rate of perceived exertion (RPE) [23] and (iii) countermovement jumps (CMJs) to evaluate the lower limbs’ anaerobic power. Blood lactate concentration was measured using a portable Lactate Pro system (KDK Corporation, Kyoto, Japan). This device has previously been validated with a great reliability (coefficients of variation ranging from 4.3 to 5%) [24]. Briefly, 5-μL blood samples were collected by capillary action using coded reagent strips and then analyzed with an amperometric method. Samples were drawn from the fingertip with the site standardized for each participant. Two minutes of passive recovery were respected between warm-up and blood lactate concentration measurement to avoid any “warm-up” effect.

CMJs were performed starting from a standing position, squatting down to a 90° knee angle (± 5°) and then extending the knees in one continuous movement. During CMJs the arms were free to help the jump. Jump height was calculated by an Optojump system (Optojump, Microgate, Bolzano, Italy) measuring the flight time of the jumps with an accuracy of 1/1000 s (1 kHz). Jump height was then estimated as 9.81 × flight time^2^/8 [25]. The Optojump system has been demonstrated to be valid and reliable for vertical jump performance assessments (correlation with results obtained with a forceplate: r = 0.99; 95%CI 0.098 to 0.99; *p* < 0.0001) [26].

***POST30 and POST60***: after recovery (i.e., 30 and 60 min after the end of intermittent exercise) participants warmed-up (using the same procedure as that at the beginning of the experiment), waited 2 min and underwent the previously described blood lactate concentration, perceived exertion and CMJ measurements.

### 2.4. Recovery Interventions

Following exhaustive exercise, participants underwent one of the three recovery interventions for 30 min (i.e., between POST and POST30 measurements). The interventions were (i) passive recovery (PASSIVE), (ii) CWT with light pedaling (COMB) and (iii) CWT without pedaling (CWT). During the passive recovery, participants were asked to remain seated on a chair for 30 min. During both contrast water recovery interventions participants were immersed underwater in a sitting position to the level of the waist for 21 min, alternating immersion at 15 °C and 37 °C every 90 s with a 5 s changeover [12,13,14,15,16,17,18,19,20,21,22,23,24,25,26,27]. In the COMB condition, participants had to be active (freely pedaling using a mini-pedaling device, Kettler, Schirmeck, France) when they were in the water. Immersion started 5 min after the end of POST tests and stopped 4 min before POST30 tests (time necessary for the participants to change into swimming/cycling shorts).

### 2.5. Statistical Analyses

Data are presented as mean values ± SD. We used parametric tests given that data were normally distributed (Shapiro–Wilk test) and had homogeneous variances (Levene test): differences among conditions and trials were analyzed using a two-way ANOVA, using the recovery modality as the among-participants factor and time as the within-participants factor. Then, Newman–Keuls post hoc tests were used when significant main effects or interactions were obtained. Effect sizes (η^2^) were also determined, with values of 0.2, 0.5 and above 0.8 considered to represent small, medium and large differences, respectively [28]. Statistical significance was tested and accepted at *p* < 0.05.

## 3. Results

No significant differences were observed between the three groups regarding both the participants’ characteristics and PRE measurements (*p* > 0.05). 

### 3.1. Effects of High-Intensity Intermittent Exercise

For the three groups high-intensity intermittent exercise induced significant decreases in maximal power, mean power and minimal power between the first and fourth sprints (Table 1, *p* < 0.05). There was no difference between the groups, revealing a similar amount of fatigue.

### 3.2. Lactate

**Figure 2:**
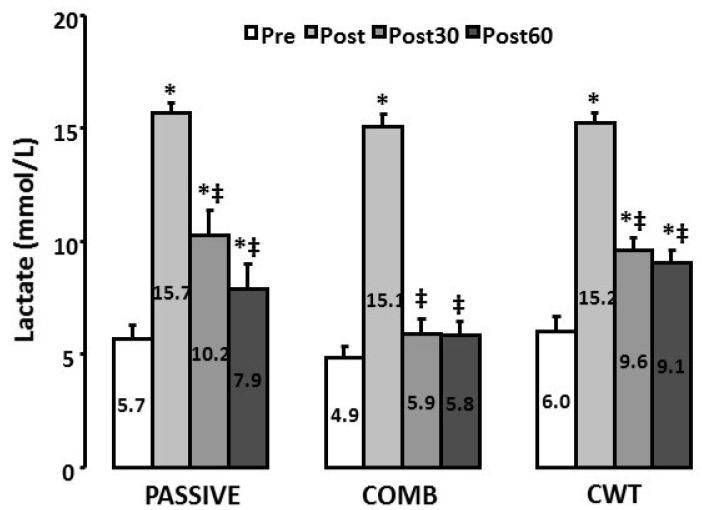
Blood lactate concentration before, immediately after, 30 min and 60 min after the exhaustive intermittent exercise. Pre: before, Post: immediately after, Post30: 30 min after, Post60: 60 min after the exhaustive intermittent exercise, PASSIVE: subjects remained passively seated, COMB: light pedalling during contrast-water immersion and CWT: subjects remained passively seated during contrast-water immersion. * Values significantly different from Pre (*p* < 0.05, Newman-Keuls Post-Hoc). ‡ Values significantly different from Post (*p* < 0.05, Newman-Keuls Post-Hoc). Values are means ± SD.

As shown in Figure 2, exhaustive exercise induced similar significant increases in blood lactate concentration for the three groups (*p* < 0.05). After 30 min of COMB recovery blood lactate concentration decreased to values not significantly different from the baseline (*p* > 0.05), whereas for the PASSIVE and CWT conditions even 60 min of recovery was not sufficient to fully recover (*p* < 0.05, η^2^ = 0.22).

### 3.3. Countermovement Jump

**Figure 3:**
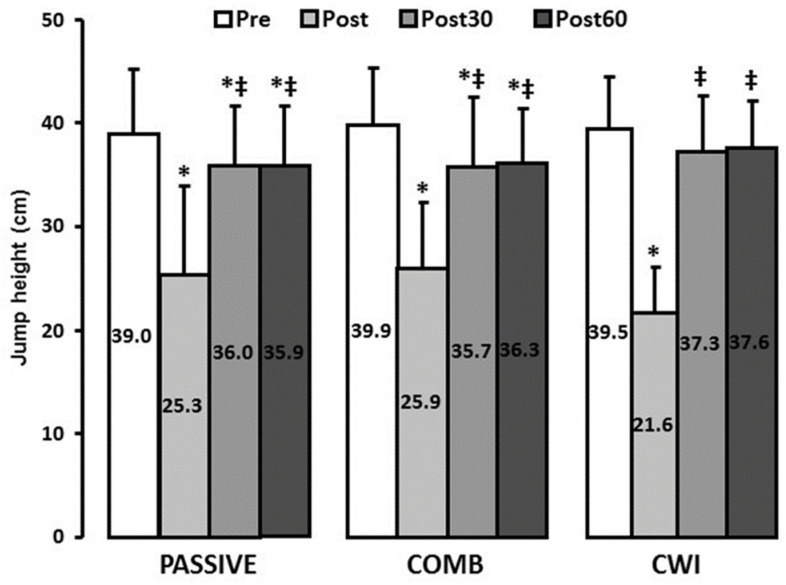
Jump height before, immediately after, 30 min and 60 min after the exhaustive intermittent exercise. Pre: before, Post: immediately after, Post30: 30 min after, Post60: 60 min after the exhaustive intermittent exercise, PASSIVE: subjects remained passively seated, COMB: light pedalling during contrast-water immersion and CWI: subjects remained passively seated during contrast-water immersion. * Values significantly different from Pre (*p* < 0.05, Newman-Keuls Post-Hoc). ‡ Values significantly different from Post (*p* < 0.05, Newman-Keuls Post-Hoc). Values are means ± SD.

Jump height was significantly decreased after exhaustive exercise (−39 ± 13% on average, *p* < 0.05), with no difference between groups. As shown in Figure 3, after 30 min of recovery in the CWI condition jump height was not significantly different from PRE values (*p* > 0.05), whereas it was still depressed after 60 min when PASSIVE or COMB recovery interventions were applied (*p* < 0.05, η^2^ = 0.20).

### 3.4. Perceived Exertion

**Figure 4:**
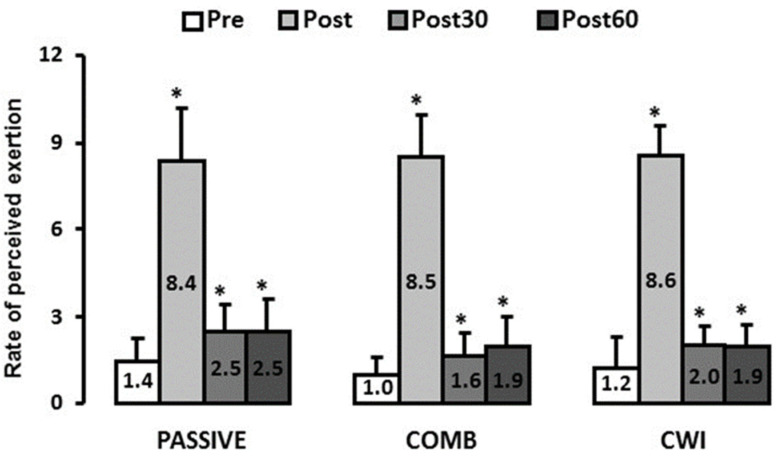
Rate of perceived exhaustion, immediately after, 30 min and 60 min after the exhaustive intermittent exercise. Pre: before, Post: immediately after, Post30: 30 min after, Post60: 60 min after the exhaustive intermittent exercise, PASSIVE: subjects remained passively seated, COMB: light pedalling during contrast-water immersion and CWI: subjects remained passively seated during contrast-water immersion. * Values significantly different from Pre (*p* < 0.05, Newman-Keuls Post-Hoc). Values are means ± SD.

As shown in Figure 4, the rate of perceived exertion was significantly increased in the three groups at the end of exhaustive exercise (*p* < 0.05, η^2^ = 0.93). Despite greater decreases after water immersion (COMB and CWT groups) these values did not return to initial values, even after 60 min, regardless of the intervention (*p* < 0.05).

## 4. Discussion

The purpose of this study was to investigate the effects of contrast water immersion used alone or combined with active recovery, as compared with passive recovery, on blood lactate concentration, jump performance and perceived exertion. We hypothesized that light pedaling added to contrast water therapy (CWT) would maximize the effects of recovery after exhaustive exercise. Our results revealed a greater clearance in blood lactate concentration in the COMB condition as compared with the CWT and PASSIVE conditions but no beneficial effect on jump performances and perceived exertion. 

It has been suggested [8] that sports that cause a large depletion in muscle energy stores or cause large increases in metabolites (high-intensity anaerobic power-endurance or endurance sports) may particularly benefit from water immersion. Indeed, the technique of CWT is thought to reproduce the muscle pumping action through alternating vasodilation (immersion in hot water) and vasoconstriction (immersion in cold water) of the blood vessels due to temperatures changes and/or hydrostatic pressure that would aid the return of fluid from the muscles into the blood [9]. However, in the present study we found that blood lactate concentration did not decrease faster after CWT than in the PASSIVE condition. This result is quite surprising and in opposition with previous data from the literature. For example, Hamlin [29] found a substantial decrease in blood lactate concentration after contrast water therapy (60 s in 8–10 °C water alternated with 60 s in 38 °C water, repeated three times) as compared with passive recovery. Although water temperatures might not be the main factor contributing to the beneficial effects observed after contrast water therapy [8], the too-small difference in cold (15 °C) and hot (37°) water temperatures might partly explain our results. In a recent review Tavares et al. [30] suggested that there might be an ideal zone for water temperature during cold water immersion (between 11 and 15 °C); Sanchez-Urena reported hot water temperatures between 38 and 42 °C [15]. Indeed, the expected “vasomotion” requires real changes (temperature, pressure) that are hardly achievable with 90 s immersion periods. For these reasons, the light pedaling added during immersion periods might have helped to create the vasomotion necessary for lactate removal. This is probably the reason why lactate decreased faster in the COMB group than in the CWT or PASSIVE groups. Similarly, several studies demonstrated that active recovery interventions (low-intensity pedaling or running) are particularly efficient in favoring lactate clearance by maintaining muscle blood flow to allow the continued efflux of lactate from active muscles in conjunction with uptake by inactive muscles [31]. In a recent study comparing several recovery interventions, Crowther et al. [13] reported enhanced recuperation of performance after active recovery in comparison to water immersion strategies. The authors suggested that these results might be due to the enhanced rate of lactate removal via quicker lactate distribution to the liver and increased lactate utilization as well as increased blood flow and accelerated recovery of interstitial creatine kinase levels.

However, our results revealed that the greatest lactate decrease in the COMB condition was not accompanied by improvements in CMJ. Surprisingly, the effects of recovery interventions on both lactate removal and subsequent performance have not been studied extensively and the results are inconclusive. Indeed, although some studies reported a concurrent decrease in blood lactate concentration and enhanced anaerobic performance after active recovery [18,27], others did not find any relationship between these two parameters [14,29]. According to Ihsan et al. [32] the preserved subsequent performance in spite of the lack of improvement in lactate clearance after water immersion suggests that the benefits of water immersion were associated with mechanisms other than alterations in local metabolic by-products. 

Another explanation could be that COMB recovery provided lesser possible benefits to the recovery process by increasing the energy requirement (for pedaling), preventing the restoration of PCr stocks and thus the performance recovery. Indeed, as suggested by Wilcock et al. [8], one of the main interests of CWT is to mimic one of the mechanisms attributed to active recovery without demanding the same amount of energy. 

This would therefore suggest that COMB recovery might not be the most appropriated method if placed between two exercises/training sessions performed close to each other. In addition, another important aspect to keep in mind while scheduling training and recovery is that lactate can be a key factor for exercise-induced mitochondrial adaptations, and that the efficacy of high-intensity training is, at least partly, attributed to elevated blood lactate concentration [33]. 

Our results also demonstrated that, despite greater decreases in the two groups who underwent water immersion, perceived exertion was still high for all participants even after 60 min of recovery. Several studies reported great effects of water immersion strategies on perceptual recovery [13,34]. For example, Crowther et al. [13] reported a better perception of recovery after COMB (cold immersion + pedaling) as compared with other recovery modalities. The authors suggested that this result might be attributable to the rapid post-exercise cooling. One could therefore argue that water temperature is an important parameter. However, similarly to our results, Vaile et al. [12] found that both cold and hot water immersion were ineffective in reducing the perception of exertion following eccentric exercise. Marcora and Bosio [35] also reported that the increase in perceived exertion with exercise-induced muscle damage was correlated with decreased 30-min time trial performance, suggesting that reductions in endurance running performance might be mediated by a “sense” of effort. Although the intermittent exercise performed in the present study probably did not induce muscle damage it was very exhausting, as demonstrated by the Borg scale’s scores and great increases in blood lactate concentration. The “sense of effort” theory might therefore explain, at least in part, our results.

This study presents some limitations that need to be noted. First, the fatiguing protocol used in the present study was chosen because it is known to increase blood lactate concentration [19]. We acknowledge that this choice might be far from the fatigue induced by team sports. Second, participants from the PASSIVE group remain seated during the 15-min recovery while participants from the COMB and CWT groups had to climb up and down from the bath, potentially adding some fatigue (increasing energy expenditure). Third, a fourth group of participants undergoing active recovery (light pedaling on land) is missing.

### Practical Applications

-If subsequent exercise is scheduled shortly after recovery, CWT should be preferred.

-If subsequent exercise/training is scheduled some time away from the initial one, adding pedaling to CWT might be of interest.

-Paying attention to water temperature is important (ideally, cold water should be between 11 and 15 °C and hot water should be between 38 and 42 °C).

## 5. Conclusions

Although water immersion interventions (COMB and CWT) may assist recovery to a greater degree than PASSIVE recovery following high-intensity exercise, they must be used according to what comes next (subsequent exercise scheduled in the following hours or some time away from the initial one). 

Further studies with more participants and matching the duration of recovery interventions are needed in order to confirm the present results and increase their statistical power. Moreover, in a further study it would be of interest to quantify the work completed during active recovery and to replicate the protocol with cold water immersion + pedaling.

## Figures and Tables

**Table 1:** Maximal, minimal and mean power values (in watts) measured during the first and fourth sprints of high-intensity intermittent exercise.

		PASSIVE	COMB	CWT
**Pmax**	Sprint 1	730 ± 123	714 ± 129	712 ± 131
	Sprint 4	554 ± 105 *	559 ± 89 *	542 ± 85 *
**Pmin**	Sprint 1	475 ± 90	478 ± 77	486 ± 61
	Sprint 4	365 ± 81 *	370 ± 61*	362 ± 44 *
**Pmean**	Sprint 1	571 ± 123	582 ± 89	573 ± 67
	Sprint 4	437 ± 87 *	450 ± 66 *	432 ± 49 *

Pmax: maximal power developed during the 30-s sprint; Pmin: minimal power developed during the 30-s sprint; and Pmean: average power developed during the 30-s sprint. PASSIVE: participants remained passively seated; COMB: light pedaling during contrast water immersion; and CWT: participants remained passively seated during contrast water immersion. * Significantly different from Sprint 1 (*p* < 0.05, Newman–Keuls post hoc).

## Data Availability

The raw data supporting the conclusions of this article will be made available by the authors, without undue reservation.

## References

[1] Reilly T., Ekblom B. (2005). The use of recovery methods post exercise. J. Sports Sci..

[2] Gill N., Beaven C., Cook C. (2006). Effectiveness of post-match recovery strategies in rugby players. Br. J. Sports Med..

[3] Martin V., Millet G., Lattier G., Perrod L. (2004). Effects of Recovery Modes after Knee Extensor Muscles Eccentric Contractions. Med. Sci. Sports Exerc..

[4] Pournot H., Bieuzen F., Duffield R., Lepretre P.-M., Cozzolino C., Hausswirth C. (2010). Short term effects of various water immersions on recovery from exhaustive intermittent exercise. Eur. J. Appl. Physiol..

[5] Tessitore A., Meeusen R., Cortis C., Capranica L. (2007). Effects of Different Recovery Interventions on Anaerobic Performances Following Preseason Soccer Training. J. Strength Cond. Res..

[6] Crowther F., Sealey R., Crowe M., Edwards A., Halson S. (2017). Team sport athletes’ perceptions and use of recovery strategies: A mixed-methods survey study. BMC Sports Sci. Med. Rehabil..

[7] Bezuglov E., Lazarev A., Khaitin V., Chegin S., Tikhonova A., Talibov O., Gerasimuk D., Waśkiewicz Z. (2021). The Prevalence of Use of Various Post-Exercise Recovery Methods after Training among Elite Endurance Athletes. Int. J. Environ. Res. Public Health.

[8] Wilcock I.M., Cronin J.B., Hing W.A. (2006). Physiological response to water immersion: A method for sport recovery?. Sports Med..

[9] Hing W.A., White S.G., Bouaaphone A., Lee P. (2008). Contrast therapy–A systematic review. Phys. Ther. Sport.

[10] Bailey D.M., Erith S.J., Griffin P.J., Dowson A., Brewer D.S., Gant N., Williams C. (2007). Influence of cold-water immersion on indices of muscle damage following prolonged intermittent shuttle running. J. Sports Sci..

[11] Vaile J., Halson S., Gill N., Dawson B. (2008). Effect of cold water immersion on repeat cycling performance and thermoregulation in the heat. J. Sports Sci..

[12] Vaile J., Halson S., Gill N., Dawson B. (2007). Effect of Hydrotherapy on Recovery from Fatigue. Int. J. Sports Med..

[13] Crowther F., Sealey R., Crowe M., Edwards A., Halson S. (2017). Influence of recovery strategies upon performance and perceptions following fatiguing exercise: A randomized controlled trial. BMC Sports Sci. Med. Rehabil..

[14] Ahokas E.K., Ihalainen J.K., Kyröläinen H., Mero A.A. (2019). Effects of Water Immersion Methods on Postexercise Recovery of Physical and Mental Performance. Strength Cond. Res..

[15] Sanchez-Urena B., Barrantes-Brais K., Urena-Bonilla P., Calleja-Gonzalez J., Ostojic S. (2015). Effect of water immersion on recovery from fatigue: A meta-analysis. Eur. J. Human Mov..

[16] Signorile J.F., Tremblay L.M., Ingalls C. (1993). The Effects of Active and Passive Recovery on Short-Term, High Intensity Power Output. Can. J. Appl. Physiol..

[17] Bogdanis G.C., Nevill M.E., Lakomy H.K., Graham C.M., Louis G. (1996). Effects of active recovery on power output during repeated maximal sprint cycling. Eur. J. Appl. Physiol. Occup. Physiol..

[18] Barnett A. (2006). Using recovery modalities between training sessions in elite athletes: Does it help?. Sports Med..

[19] Ztürk M., Özer K., Gökçe E. (1998). Evaluation of blood lactate in young men after wingate anaerobic test. East. J. Med..

[20] Laurent C.M., Meyers M.C., Robinson C.A., Green J.M. (2007). Cross-validation of the 20- versus 30-s Wingate anaerobic test. Eur. J. Appl. Physiol..

[21] Morton R.H. (2007). Contrast water immersion hastens plasma lactate decrease after intense anaerobic exercise. J. Sci. Med. Sport.

[22] Olek R., Ziemann E., Grzywacz T., Kujach S., Luszczyk M., Antosiewicz J., Laskowski R. (2010). A single oral intake of arginine does not affect performance during repeated Wingate anaerobic test. J. Sports Med. Phys. Fit..

[23] Borg G., Borg G., Ottoson D. (1986). Psychophysical Studies of Effort and Exertion: Some Historical, Theoretical and Empirical Aspects. The Perception of Exertion in Physical Work.

[24] Tanner R.K., Fuller K.L., Ross M.L.R. (2010). Evaluation of three portable blood lactate analysers: Lactate Pro, Lactate Scout and Lactate Plus. Eur. J. Appl. Physiol..

[25] Bosco C., Luhtanen P., Komi P.V. (1983). A simple method for measurement of mechanical power in jumping. Eur. J. Appl. Physiol. Occup. Physiol..

[26] Castagna C., Ganzetti M., Ditroilo M., Giovannelli M., Rocchetti A., Manzi V. (2013). Concurrent Validity of Vertical Jump Performance Assessment Systems. J. Strength Cond. Res..

[27] Heyman E., DE Geus B., Mertens I., Meeusen R. (2009). Effects of Four Recovery Methods on Repeated Maximal Rock Climbing Performance. Med. Sci. Sports Exerc..

[28] Cohen J. (1988). Statistical Power Analysis for the Behavioural Sciences.

[29] Hamlin M.J. (2007). The effect of recovery modality on blood lactate removal and subsequent repetitive sprint performance in netball players. N. Z. J. Sports Med..

[30] Tavares F., Walker O., Healey P., Smith T.B., Driller M. (2018). Practical Applications of Water Immersion Recovery Modalities for Team Sports. Strength Cond. J..

[31] Monedero J., Donne B. (2000). Effect of Recovery Interventions on Lactate Removal and Subsequent Performance. Int. J. Sports Med..

[32] Ihsan M., Watson G., Abbiss C.R. (2016). What are the Physiological Mechanisms for Post-Exercise Cold Water Immersion in the Recovery from Prolonged Endurance and Intermittent Exercise?. Sports Med..

[33] Takahashi K., Kitaoka Y., Matsunaga Y., Hatta H. (2019). Effects of lactate administration on mitochondrial enzyme activity and monocarboxylate transporters in mouse skeletal muscle. Physiol. Rep..

[34] Ingram J., Dawson B., Goodman C., Wallman K., Beilby J. (2009). Effect of water immersion methods on post-exercise recovery from simulated team sport exercise. J. Sci. Med. Sport.

[35] Marcora S.M., Bosio A. (2007). Effect of exercise-induced muscle damage on endurance running performance in humans. Scand. J. Med. Sci. Sports.

